# High-sensitivity virus and mycoplasma screening test reveals high prevalence of parvovirus B19 infection in human synovial tissues and bone marrow

**DOI:** 10.1186/s13287-018-0811-7

**Published:** 2018-03-27

**Authors:** Ken Watanabe, Koji Otabe, Norio Shimizu, Keiichirou Komori, Mitsuru Mizuno, Hisako Katano, Hideyuki Koga, Ichiro Sekiya

**Affiliations:** 1 Virus Research Unit, Center for Stem Cell and Regenerative Medicine, Tokyo Medical and Dental University, Tokyo, Japan; 2 Center for Stem Cell and Regenerative Medicine, Tokyo Medical and Dental University, 1-5-45 Yushima, Bunkyo-ku, Tokyo, 113-8519 Japan; 3 Department of Orthopaedic Surgery, Tokyo Medical and Dental University, Tokyo, Japan

**Keywords:** Mesenchymal stem cells, Synovium, Bone marrow, Infection screening, Virus, Mycoplasma

## Abstract

**Background:**

Latent microorganism infection is a safety concern for the clinical application of mesenchymal stem cells (MSCs). The aim of this study is to investigate the frequencies and sensitivities of the latent virus and mycoplasma infections in synovium, bone marrow, peripheral blood cells, and blood plasma and cultured synovial MSCs.

**Methods:**

Total DNA and RNA of the synovium (*n* = 124), bone marrow (*n* = 123), peripheral blood cells (*n* = 121), plasma (*n* = 121), and 14-day cultured synovial MSCs (*n* = 63) were collected from patients who underwent total knee arthroplasty or anterior ligament reconstruction after written informed consents were obtained. The multiplex polymerase chain reaction (PCR) primers were designed to quantitatively measure the representative genomes of 13 DNA viruses, 6 RNA viruses, and 9 mycoplasmas. Multi-spliced mRNA detection and virus spike test were also performed to demonstrate the sensitivity of synovial MSCs to the candidate pathogens.

**Results:**

In synovium and bone marrow, the positive rates of parvovirus B19 genome were significantly higher than in peripheral blood cells (18.7% and 22% vs. 0.8%, respectively). Multi-alignment analysis of amplified and sequenced viral target genes showed the proximity of the parvovirus B19 gene from different tissue in the same patients. Synovial MSCs cultured for 14 days were positive for virus infection only in two patients (2/62 = 3%). Parvovirus B19 multi-spliced mRNAs were not detected in these two samples. Virus spike test demonstrated the sensitivity of synovial MSCs to herpes simplex virus (HSV)1 and cytomegalovirus (CMV), but not to parvovirus B19.

**Conclusion:**

This study revealed a relatively high incidence of latent parvovirus B19 in synovium and bone marrow tissue.

**Electronic supplementary material:**

The online version of this article (10.1186/s13287-018-0811-7) contains supplementary material, which is available to authorized users.

## Background

Osteoarthritis (OA) is characterized by degenerative locomotive structures such as cartilage, bone, synovium, meniscus, ligament, and tendon tissues. OA is often accompanied by severe pain and morbidity and now places enormous economic and sociologic burden on the worldwide healthcare provider [1]. OA is not a simple joint disease and it is considered to be brought about by a number of systemic factors (age, gender, obesity, inactivity, genetics, and so on) and mechanical factors (obesity, joint morphology and alignment, joint loading, muscle weakness, injury, and so forth) [2]. A truly multidisciplinary effort has been aimed at the prevention and treatment of OA, but its complex background and pathophysiology hinder the development of a definitive coping technique.

The cartilage and meniscus are very vulnerable tissues in the osteoarthritic joints and have a poor regenerative property because of the absence of any vasculature and their sparse cellular components compared to the rich intercellular matrix [3]. Regenerative therapies for cartilage and meniscus injury using stem cells have been highly envisaged. Mesenchymal stem cells (MSCs) possess a self-replication property, a multilineage differentiation capacity, and a relatively low risk of tumorigenesis compared to induced pluripotent stem cells (iPSCs). Promising MSC sources are bone marrow [4, 5], adipose tissue [6], muscle tissue [7], and the synovium [8]. Of these candidates, the synovium is known to harbor highly chondrogenic MSCs [8, 9] and shows virtually no donor site morbidity because of the abundance and regenerative property of the synovial tissue. Currently, and supported by the abundant evidence from animal studies [10–16], clinical trials with synovial MSCs cultured with autoserum for intractable cartilage and meniscus injury are being performed [17].

Latent virus and mycoplasma infection in the stem cell sources is one of the safety concerns for any clinical application. For the purpose of ensuring safety, a high-sensitivity, broad-range, real-time polymerase chain reaction (PCR) system to detect latent virus and mycoplasma infections has been developed [18–22]. These studies showed good accord with clinical findings and immunohistological testing, and demonstrated high accuracy and precision.

We have applied this multiplex qualitative PCR for the prospective cell source (synovium and bone marrow) and autoserum for joint regenerative therapies. Our results suggest that mycoplasmas were not present in these tissues, but several viral species, especially parvovirus B19, Epstein-Barr virus (EBV), and human herpes virus (HHV)6, showed unexpectedly higher incidences of latent infection than others indicating a potential hazard for their use in regenerative therapy.

The aim of this study was to investigate and compare the frequencies of the virus and mycoplasma infections in several tissues for feasible cell and tissue sources and to demonstrate the efficiency of this established multiplex PCR system as a screening test for stem cell therapy in locomotor apparatuses.

## Methods

### DNA and RNA isolation, and detection of viruses

Forty-two anterior cruciate ligament (ACL) reconstruction surgery candidates (average age 28 ± 11 years old, male:female ratio 23:19; a total of 42 knees) and 88 total knee arthroplasty (TKA) candidates (average age 74 ± 8 years old, male:female ratio 21:67; six patients underwent simultaneous bilateral TKA, thus a total of 94 knees) were enrolled in this study. Synovium (*n* = 123), bone marrow (*n* = 122), peripheral blood cells (*n* = 120), plasma (*n* = 120), and 14-day cultured synovial MSCs (*n* = 62) were collected from patients who underwent ACL reconstruction surgery or TKA after written informed consents were obtained.

Total DNA was extracted from solid and cellular samples using a QiAamp DNA minikit (Qiagen, Valencia, CA, USA) and total RNA was collected by the RNeasy mini kit (Qiagen). QIAamp MinElute Virus Spin Kit (Qiagen) was applied for liquid samples.

We designed a seven-tube multiplex for detection of 13 DNA viruses (human herpes simplex virus (HSV)1, HSV2, human hepatitis B virus (HBV), BK virus (BKV), human polyomavirus (JCV), EBV, varicella zoster virus (VZV), HHV6, HHV7, HHV8, adenovirus (ADV), cytomegalovirus (CMV), and parvovirus B19), and six-tube multiplex for detection of six RNA viruses (human immunodeficiency virus (HIV)1, HIV2, human T-cell leukemia virus (HTLV)1, HTLV2, West Nile virus (WNV), and human hepatitis C virus (HCV)). These products were manufactured, entrusted to the Nihon techno service corporation (thirteen DNA viruses: NT1202-MP DNA virus strip ver. 8.5 12 pcs/pack; six RNA viruses: NT1303-RMG-MP-RNA virus strip ver. KW1505 12 pcs/pack; Additional file 1: Figure S1). The DNA viruses were amplified by quantitative PCR (qPCR) using DNA virus strip and 0.1 U taq DNA polymerase (Thermo), anti-taq high (TOYOBO), 3 mM MgCl_2_, and 1 mM dNTPs (genscript). Total volumes were adjusted to 20 μL on a CFX96 (Bio-Rad) and underwent qPCR with the following cycling conditions: 95 °C for 10 s, 45 cycles (95 °C for 5 s, 60 °C for 30 s). The RNA viruses were amplified by RT-qPCR using RNA virus strip and One step PrimeScript RT-PCR kit (Perfect Real time) (TaKaRa-Bio). Total volumes were adjusted to 20 μL on a LightCycler DX480 (Roche) and underwent qPCR with the following cycling conditions: 42 °C for 5 min, 95 °C for 10 s, 45 cycles (95 °C for 5 s, 60 °C for 30 s). Primer and probe sequences are shown in Additional file 2 (Table S1).

### Quantitative PCR detection of mycoplasma species

The mycoplasma genomic DNA was amplified by qPCR using primers and probes and 0.25 U taq DNA polymerase (Thermo), anti-taq high (TOYOBO), 3 mM MgCl_2_, and 1 mM dNTPs (genscript). Total volumes were adjusted to 50 μL on a LightCycler DX480 (Roche) and underwent qPCR with the following cycling conditions: 95 °C for 10 s, 45 cycles (95 °C for 5 s, 60 °C for 1 min). This test covers 142 mycoplasma species including 9 mycoplasmas (*M. orale*, *M. arginine*, *M. fermentans*, *M. hyorhinis*, *M. pneumoniae*, *M. synoviae*, *M. gallisepticum*, *A. laidlawii*, and *S. citri*). Primer and probe sequences are shown in Additional file 2 (Table S1).

### Parvovirus B19 phylogenetic analysis

The target parvovirus B19 gene (NS1-VP1u) from the parvovirus B19-positive samples (*n* = 26) were amplified by semi-nested PCR using primers and 2 U PrimeSTAR GXL DNA polymerase (TaKaRa-Bio), 1× PrimeSTAR GXL Buffer, and 0.8 mM dNTPs (TaKaRa-Bio) on a CFX96 (Bio-Rad) with the following 1st and 2nd cycling conditions: 98 °C for 10 s, 45 cycles (98 °C for 5 s, 60 °C for 0 s, 68 °C 60s); final volumes of all samples were 50 μL and the product was sequenced. (Additional file 3: Table S2).

The data were analyzed by Clustal W software [23] and a phylogenetic image was made by MEGA 5.2 software (http://www.megasoftware.net). The magnitude of the difference of the nucleotide sequence was analyzed in accord with the Kimura two-parameter distance model [24]. Bootstrap values over 75% are shown.

### Virus spike tests for synovial MSCs

#### Cells and viruses

HSV1 (strain F) was propagated on Vero cells. Human CMV (HCMV; Towne) was propagated on human fetal lung fibroblast cells (HFL-1 cells; Riken Cell Bank, Tsukuba, Japan). Parvovirus B19-positive serum was used. KU812Ep6 cell line culture and virus titration of parvovirus B19-positive serum were performed according to the described method [25]. HFL-1 cells and Vero cells were grown in Eagle’s minimum essential medium (MEM; Gibco) supplemented with 10% fetal bovine serum (FBS; Gibco) and HFL-1 cells were maintained in MEM supplemented with 2% FBS. Titration of HCMV was determined as plaque-forming units by the agarose overlay method [26].

Titration of HSV1 was determined as plaque-forming units by the agarose overlay method. Briefly, duplicate cultures of Vero cells were prepared in a six-well plate and 1 mL of virus suspension was added after removal of maintenance medium. The inoculum was aspirated after 1 h of adsorption at 37 °C in a 5% CO_2_ air atmosphere, and the cultures were washed once with approximately 2 mL of MEM supplemented with 2% FBS and overlaid with 2 mL of MEM supplemented with 2% FBS containing 1.5% agarose at 37 °C. Plaques were counted by microscope without staining 2 days after infection.

Synovial MSCs were obtained using the following operations. The synovium was digested in a solution of 5 mg Liberase™ (Roche) in 5 mL Hanks’ balanced salt solution (HBSS; Invitrogen, Carlsbad, CA, USA) at 37 °C for 3 h. The digested cells were filtered through a 70-μm nylon filter (Becton Dickinson). Synovial MSCs were resuspended at 1 × 10^5^/90-mm dish in MEMα-1 supplemented with 10% FBS (Gibco; FBS were irradiated 35 kGy). After 3 days, synovial MSCs were infected with viruses at a multiplicity of infection of: HSV1, 0.39 PFU/cell; HCMV, 0.35 PFU/cell; and parvovirus B19, 0.001 TCID50/cell.

#### Antibodies and immunofluorescence

Virus spiked and unspiked cultures in 12-well cell culture plates were dried for 1 h and then fixed with 1 mL of −20 °C methanol and incubated for 10 min at −20 °C and then dried for 1 h and rinsed with phosphate-buffered saline (PBS). Blocking was performed for 10 min in 400 μL blocking buffer (10% rabbit serum, 10% FBS in PBS). After blocking, the buffer cells were incubated overnight at 4 °C with the following antibodies: parvovirus B19 VP1/2 (Millipore MAB8293), HSV1/2 ICP4 (Santa Cruz biotechnology sc-56,986), HSV1/2 glycoprotein B (Santa Cruz biotechnology sc-56,987), CMV pp65 (Santa Cruz biotechnology sc-71,229), CMV glycoprotein B (Santa Cruz biotechnology sc-52,400). Parvovirus B19 VP1/2 antibodies were diluted 200-fold in the buffer (10% FBS in PBS), and others were diluted to 50-fold. Cells were washed three times with 1 mL PBS and after the last incubation step. Fluorescein isothiocyanate (FITC)-conjugated secondary antibody (polyclonal rabbit anti-mouse immunoglobulins/FITC, Dako F0261) were used for the detection of primary antibodies. Cells were incubated in the dark for 2 h at room temperature. Secondary antibody was diluted 20-fold in the buffer (10% FBS in PBS). Cells were mounted with fluorescent mounting medium (Dako) and covered with a slide glass (18 mm microcover glass, MATSUNAMI) after the washing step. The infected cells were checked by fluorescence microscopy (eclipse Ti-E, Nikon).

### Quantitative PCR analysis for viral mRNA

The three viral mRNAs (parvovirus B19, CMV, and HSV1) was amplified by RT-qPCR using three DNA viral mRNA primers and probes and PrimeScript one-step real-time RT-PCR kit (TaKaRa-Bio) on a LightCycler DX480 (Roche) with the following cycling conditions: 42 °C for 5 min, 95 °C for 10 s, 45 cycles (95 °C for 5 s, 60 °C for 30 s) (Additional file 4: Table S3 and Additional file 5: Figure S2.)

### Statistical analysis

Comparisons were performed using the Fisher’s exact with Bonferroni correction for multiple comparisons, or chi squared test. *P* values less than 0.05 were considered significant.

## Results

### Multiplex PCR analysis revealed viral infections in tissues obtained during knee operations and synovial MSCs

**Fig. 1:**
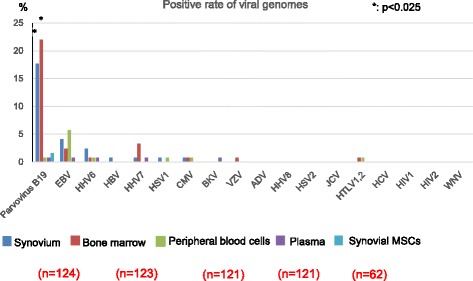
Positive rate of the viral genome in human tissues and synovial mesenchymal stem cells (MSCs). The positive rate of parvovirus B19 in the synovium and bone marrow was significantly higher than in whole blood, plasma, and synovial MSCs; *p < 0.025 by the Fisher’s exact test with Bonferroni correction for multiple comparison

The highest positive rate of viral genomes was 22% for parvovirus B19 in bone marrow (Fig. 1). Although the positive rate of parvovirus B19 in the synovium was also high (18%), 14 days after culture of synovial nucleated cells the rate decreased to 2% in synovial MSCs. Parvovirus B19 was detected in all kinds of tissues and cells we examined; however, its positive rate in bone marrow and synovium was significantly higher than in whole blood and plasma. In younger subjects (28 ± 11 years old) who underwent ACL reconstruction, the positive rate of parvovirus B19 in the synovium was 26%, significantly higher than the 14% in older subjects (74 ± 8 years old) who underwent TKA (Table 1). Other virus species detected in any tissues we examined were EBV, HHV6, HHV7, HSV1, HBV, CMV, BKV, VZV, and HTLV1/2. Virus species we did not detect in any tissues or synovial MSCs were HSV2, JCV, HHV8, ADV, HIV1, HIV2, WNV, and HCV. Mycoplasma infection was not detected in any tissues or synovial MSCs.

**Table 1:** Patient background and positive parvovirus B19 rate

	Anterior cruciate ligament	Total knee arthroplasty
Sample size	42 knees (*n* = 42; male:female = 23:19)	94 knees (*n* = 88; male:female = 21:67)
Age	28 ± 11 years	74 ± 8 years
Parvovirus B19+	11 (26%)	13 (14%)

### Parvovirus B19 phylogenetic analysis showed the existence of viral genome in different parts of the body in the same patient

**Fig. 2:**
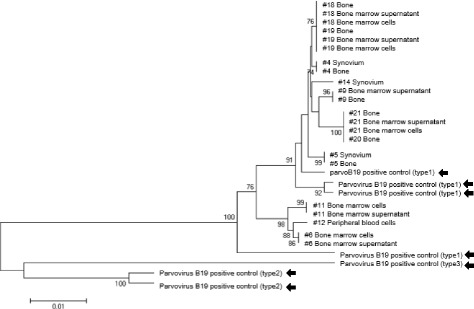
Parvovirus B19 phylogenetic analysis. The amplified viral target genes were sequenced and arranged on the basis of similarity of the sequence. Arrows show parvovirus B19 positive control reference; # number shows the donor number. Value on the phylogenetic tree represents the bootstrap values

Of the 30 samples that were positive for parvovirus B19 derived from 36 donors, 26 samples underwent multi-alignment analysis of the amplified and sequenced for viral target gene (NS1-VP1u), and 22 samples yielded successful results (Fig. 2). Genetic types of the detected parvovirus B19 were similar to the type 1 rather than type 2 or 3 in all the cases. Samples from patient numbers 18, 19, 20, and 21 yielded positive qualitative PCR results from different tissue source. Sequences of the parvovirus B19 from several tissues from the same donor aligned to the proximate place in the phylogenetic tree.

### Parvovirus B19 DNA genome was confirmed in synovial tissue and synovial MSCs, but viral mRNA was confirmed in synovium and synovial MSCs

**Fig. 3:**
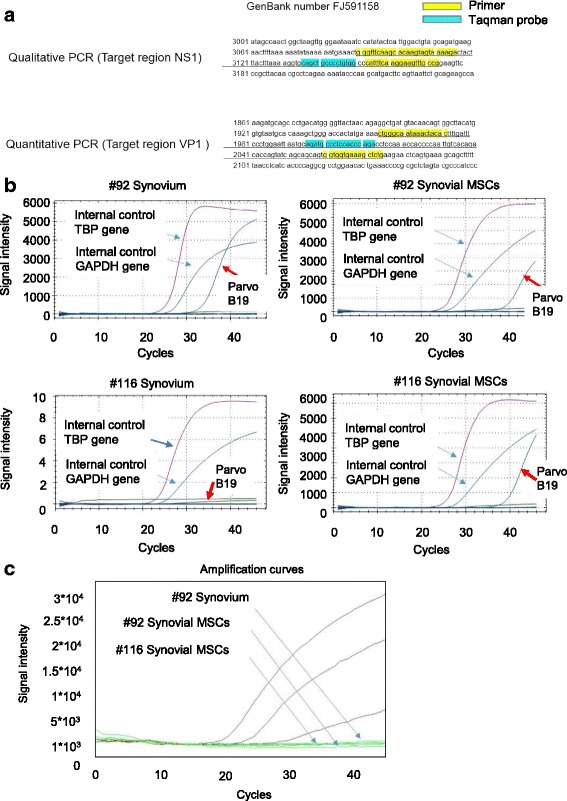
Polymerase chain reaction (PCR) analysis for parvovirus B19 genome and mRNA in synovium and synovial mesenchymal stem cells (MSCs). **a** Primer and Taqman probe sequences of qualitative and quantitative PCR for parvovirus B19 genome. **b** Signal intensity curve of qualitative PCR analysis for parvovirus B19 genome. **c** RT-PCR analysis of parvovirus B19 multi-spliced mRNA for cultured synovial MSCs

Synovial MSCs were positive for parvovirus B19 in only two patients out of 62 (2/62 = 3%) (Fig. 1). To confirm this result, primers for quantitative PCR for the target gene were designed at a different site to the first PCR analysis (Fig. 3a). The quantitative PCR showed that one patient (#116) showed a negative result for synovial tissue (Fig. 3b). Synovium in another patient (#92) and synovial MSCs in both patients still showed positive results for parvovirus B19.

The viability and reproductivity of the detected parvovirus B19 in synovial MSCs remained unknown. To answer this question, we tried to detect parvovirus B19 transcribed mRNAs. Primer and probes were designed to detect parvovirus B19 multi-spliced mRNAs to increase the power of analysis (Additional file 5: Fig. S2a). The parvovirus B19 multi-spliced mRNAs were not detected in the synovial tissues or the cultured synovial MSCs in either patient (Fig. 3c).

### Virus spike test revealed that parvovirus B19 had no infectiveness to synovial MSCs

**Fig. 4:**
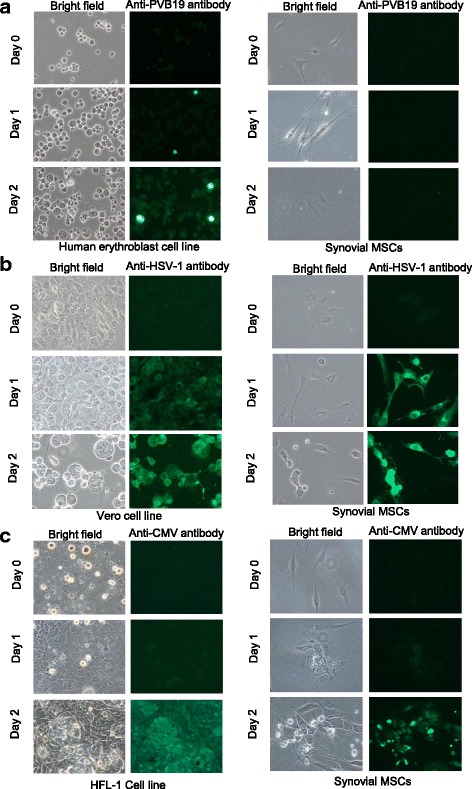
Virus spike test for synovial mesenchymal stem cells (MSCs). **a** Parvovirus B19 spike test (PVB19 VP1/2 antibody; 1:200 dilution). **b** Herpes simplex virus (HSV) spike test (HSV1/2 gB antibody (10B7); 1:50 dilution). **c** Cytomegalovirus (CMV) spike test (CMV pp65 antibody (1B228); 1:50 dilution). Left two columns represent positive control cells sensitive to each virus species and right two columns represent synovial MSCs. Viral protein was demonstrated by immunofluorescence stain

**Fig. 5:**
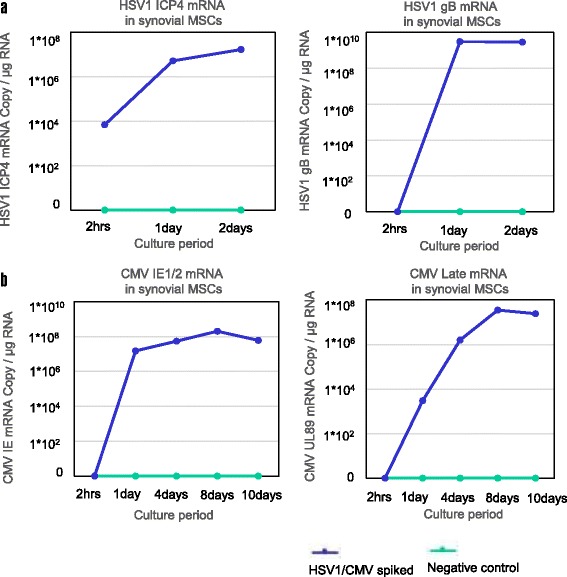
Quantitative RT-PCR analysis of virus spike test for synovial mesenchymal stem cells (MSCs). **a** Herpes simplex virus 1 (HSV1) spike test for synovial MSCs. **b** Cytomegalovirus (CMV) spike test for synovial MSCs

To test the pathogenicity of the detected virus species, we performed the viral spike test for cultured synovial MSCs. A cytopathic effect (CPE) was not confirmed for parvovirus B19, but HSV1 and CMV caused CPE on synovial MSCs and viral protein transcription was detected by immunofluorescent stain (Fig. 4). HSV1 and CMV mRNAs were also detected and increased in accord with the culture periods (Fig. 5).

## Discussion

The clearance of infective organisms from the cells to be used for stem cell therapy is of pivotal importance but there have been only sporadic studies on the latent infective organisms in mesenchymal tissues derived from the knee joint. This study showed that our established multiplex PCR and real-time PCR analysis system allowed the detection of the clinically occult microorganism infections and demonstrated an unexpectedly high incidence and broad spectrum of latent viral infections in synovium and bone marrow tissues. Both these tissues are being utilized more and more as the optimal cell sources for regenerative medicine [17, 27–31] and, therefore, this study alerts us that we have to scrutinize the safety and efficacy of the possibly infected MSCs more carefully. In fact, we found the existence of seven types of herpes viruses (HSV1, HSV2, VZV, EBV, CMV, HHV6, and HHV7), parvovirus B19, and HTLV1/2 in the synovium, bone marrow, peripheral blood, and cultured synovial MSCs in ACL and TKA patients. In contrast, mycoplasmas were not detected in all the samples. These results suggest the existence of the viral genomes in synovium, bone marrow, peripheral blood cells, blood plasma, and cultured synovial MSCs in both young (ACL reconstruction candidates) and old (TKA candidates) patients, even if they showed no apparent symptom of virus infection. To our knowledge, this is the first study to screen the encompassing, flagship microorganisms by high-sensitivity, comprehensive multiplex and real-time PCR analyses in synovium and bone marrow tissues.

We confirmed a high incidence of parvovirus B19 contaminations in the synovium and bone marrow tissue. Parvovirus B19 is a small, non-enveloped, icosahedral DNA virus, transmitted via the respiratory tract and is the classic cause of fifth disease or erythema infectiosum in childhood, abortion due to hydrops fetalis, or transient arthritis in the adult [32]. Parvovirus B19 has the capability of invading red blood cell precursors in the bone marrow and sometimes brings about aplastic anemia among the sickle cell disease or spherocytosis. Parvovirus B19 is also associated with transient, chronic, and recurrent symmetrical polyarthritis mainly affecting the small joints of the upper extremity [33–35]. Parvovirus B19 is thought to have latent and persistent infectivity [36]. Women tend to be susceptive to parvovirus B19 arthritis according to former reports [33, 34], but our results showed no significant difference between the sexes in the synovium of ACL and TKA (ACL, male:female ratio 6/18 (33%):4/16 (25%); TKA, male:female ratio 5/22 (14%):18/68 (15%)). Another study also pointed out that parvovirus B19 is detected by PCR more frequently in rheumatoid arthritis (RA) patients than in OA or other arthritic patients (75% vs. 16.7%) [36]. We did not include RA patients in this study, but the positive rate of parvovirus B19 in OA patients is very similar to this study.

The parvovirus gene multi-alignment analysis result showed that the same type of viral genome exists in different parts of the body in the same patient at the time of sample harvest. Thus, the mode of parvovirus B19 infection to the synovium seems to be the consequence of the viremia secondary to the infection of the bone marrow hematopoietic precursors, which is the classic target of the parvovirus B19.

Relatively high incidences of parvovirus B19 in the synovium and bone marrow tissues were demonstrated. In rare cases, very small copies of viral genome were still detected in the cultured synovium MSCs, but viral mRNAs could not be detected suggesting that the parvovirus B19 in cultured synovial MSCs seems to be inactive and unable to proliferate. Virus spike test results for synovial MSCs also showed that synovial MSCs have virtually no sensitivity to parvovirus B19. A recent study has demonstrated Epstein-Barr virus infection of autoreactive plasma cells in synovial lymphoid structures in RA [37]. To prepare synovial MSCs, we employed the bulk culture method [17], which means that synovial MSCs are cultured in a mixture of synovial fibroblasts, vascular pericytes, and lymphoid tissue cells in the synovium. Parvovirus B19 seems to mainly infect these types of cells rather than synovial MSCs.

RA and microorganism infection has been discussed in several studies [38–40]. A recent study suggests the cross-reactivity to autoantigen and microbes contributes to the pathogenesis of anti-citrullinated protein antibody-positive RA and its close, HLA-based protection [41]. By a similar mechanism, other types of inflammatory joint pathology of unknown cause could be triggered by the overlooked infections of the microorganisms. This study has opened the path to find such candidate “subclinical” organisms, and the multiplex PCR system will lead to the development of novel and powerful diagnostic and therapeutic decision-making tools.

The virus spike test results also suggested that parvovirus B19 has no affinity to synovial MSCs, but HSV1 and CMV could invade and proliferate in the synovial MSCs if they had a high enough titer. On the other hand, according to the result of the screening test for TKA and ACL patients, HSV1 genome was detected from one TKA patient, and CMV genome was detected from another TKA patient. But both viruses were not detected from their cultured synovial MSCs. These discrepant results may indicate the contamination of the patients’ blood cells in the synovial tissue at the occult or convalescence stage of the HSV1 or CMV systemic disease. It seems that the small fragment of the viral genomes existed in the synovial tissue of these TKA patients, but the viruses did not have enough cytopathic activity or had less than minimum titer to invade and proliferate in the synovial MSCs against the host immunity.

Worldwide, health regulatory authorities require human tissue used for regenerative medicine to be clear of viral infection. According to the many guidelines for clinical trials with human stem cells, viral species highly profiled in regenerative medicine are mainly blood-borne viruses with persistent infection, and parvovirus B19, HSV1, and CMV are included in this category. Before acquisition of human stem cells for transplantation, regulatory officials demand that these organisms be excluded by medical testing, including serologic testing or nucleic acid amplification testing. In many cases, the viral titer is too small to establish disease transmission but, as the results of the viral spike test suggest, the possibility of viral amplification during the process of cell culture still exists if viral contamination is overlooked at the initial screening. Furthermore, even if the nature of the organism is not so virulent, viral infection of the MSCs sometimes impairs the proliferative and differentiation potential of MSCs and may lead to failure of the cell therapy. This background justifies the high-sensitivity PCR screening system to rule out infection of these organisms before cell therapy for cartilage and meniscus repair is performed.

## Conclusion

In conclusion, we found the latent virus infection, such as HSV1, EBV, HHV6, HHV7, CMV, HTLV1/2, and parvovirus B19, in the synovium and bone marrow among both young and old populations. In this study, we demonstrated the usefulness of the multiplex PCR system for detecting latent viral infection in synovium and bone marrow.

## Additional files

Additional file 1: **Figure S1.** Design of DNA virus strip and RNA virus strip for multiplex qualitative polymerase chain reaction (PCR) kit. (PPTX 58 kb)

Additional file 2: **Table S1.** Primer and probe sequences employed in multiplex qualitative polymerase chain reaction (PCR) analyses. (DOCX 27 kb)

Additional file 3: **Table S2.** Primer and probe sequence of nested PCR analysis and sequencing for parvovirus B19 virus genome. (DOCX 14 kb)

Additional file 4: **Table S3.** Primer and probe sequence of nested PCR analysis and sequencing for HSV-1, CMV, and parvovirus B19 mRNAs. (DOCX 14 kb)

Additional file 5: **Figure S2.** Primer and probe design for quantitative PCR analysis for virus multi-spliced mRNA. (A) Parvovirus B19. (B) CMV. (C) HSV-1. (PPTX 135 kb)

## Abbreviations

ACL: Anterior cruciate ligament
ADV: Adenovirus
BKV: BK virus
CMV: Cytomegalovirus
CPE: Cytopathic effect
EBV: Epstein-Barr virus
FBS: Fetal bovine serum
HBSS: Hanks’ balanced salt solution
HBV: Hepatitis B virus
HCV: Hepatitis C virus
HHV: Human herpes virus
HIV: Human immunodeficiency virus
HMCV: Human cytomegalovirus
HSV: Herpes simplex virus
HTLV: Human T-cell leukemia virus
iPSC: Induced pluripotent stem cell
JCV: Human polyomavirus
MEM: Minimum essential medium
MSC: Mesenchymal stem cell
OA: Osteoarthritis
PBS: Phosphate-buffered saline
PCR: Polymerase chain reaction
RA: Rheumatoid arthritis
TKA: Total knee arthroplasty
VZV: Varicella zoster virus
WNV: West Nile Virus
