# Supplementary figures and images for: High-sensitivity virus and mycoplasma screening test reveals high prevalence of parvovirus B19 infection in human synovial tissues and bone marrow
Source: Stem Cell Res Ther. 2018 Mar 27;9:80. doi: 10.1186/s13287-018-0811-7 (PMC5870688; doi:10.1186/s13287-018-0811-7)

## Slide 1
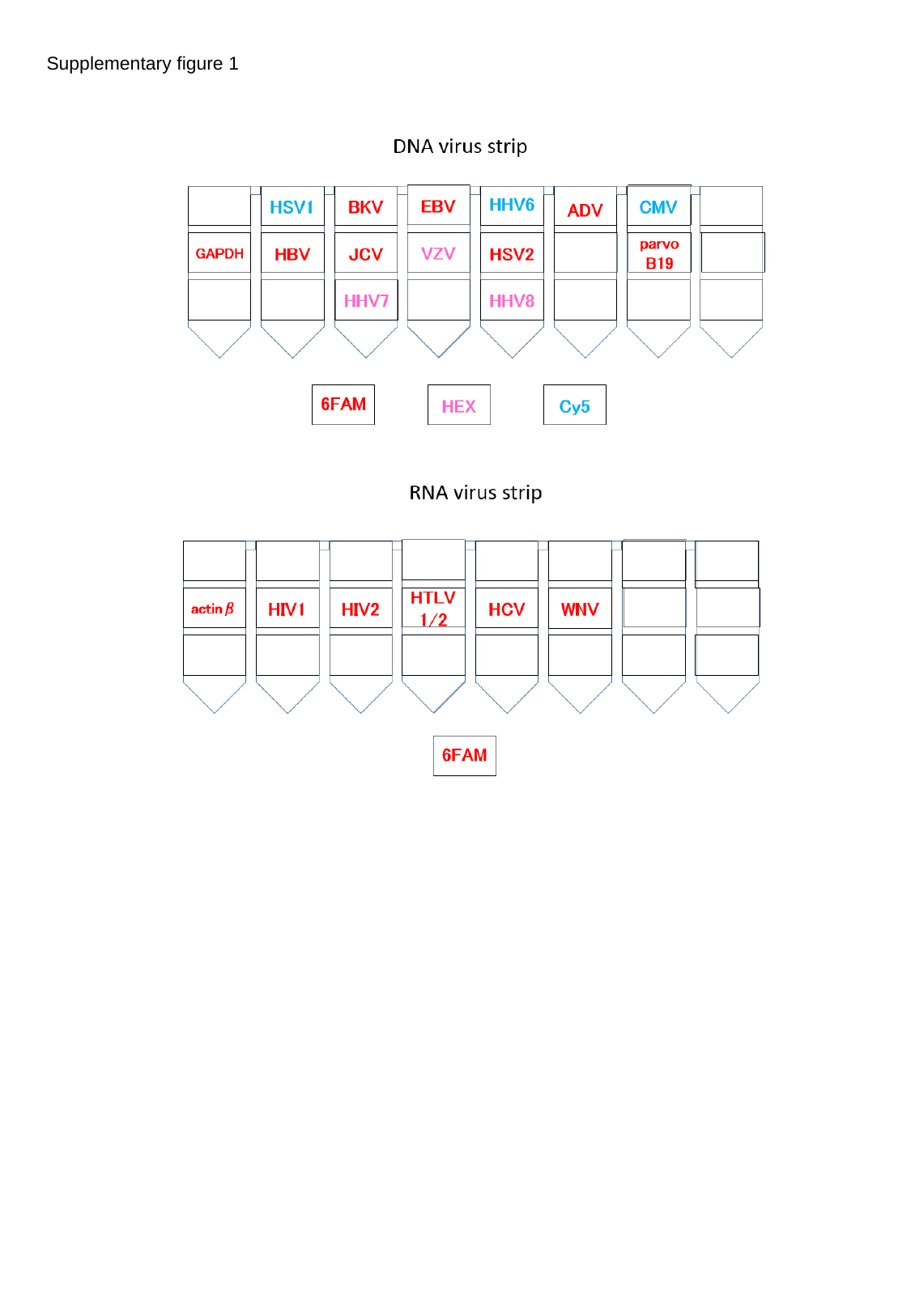

Supplementary figure 1

Supplement: Supplementary file 1 — Figure S1. Design of DNA virus strip and RNA virus strip for multiplex qualitative polymerase chain reaction (PCR) kit. (PPTX 58 kb) [file 13287_2018_811_MOESM1_ESM.pptx]
