# Supplementary material for: High-sensitivity virus and mycoplasma screening test reveals high prevalence of parvovirus B19 infection in human synovial tissues and bone marrow
Source: Stem Cell Res Ther. 2018 Mar 27;9:80. doi: 10.1186/s13287-018-0811-7 (PMC5870688; doi:10.1186/s13287-018-0811-7)
Supplement: Supplementary file 2 — Table S1. Primer and probe sequences employed in multiplex qualitative polymerase chain reaction (PCR) analyses. (DOCX 27 kb) [file 13287_2018_811_MOESM2_ESM.docx]

| Supplementary table　1.　Primer and Probe Sequences Employed in Multiplex Qualitative Polymerase Chain Reaction (PCR) Analyses | | |
| --- | --- | --- |
| DNA virus | Primer sequence | Probe sequence |
| AdenoVirus(1-3,5-8,10-12, 16,17,19,21,28,31,34,40,48) | F-gacatgacttttgaggtgga R-tcgatgacgccgcggtg | 6FAM-cccatggaygagcccaccct-BHQ |
| HBV | F–gtggtggacttctctcaattttctag R-ggacaMacgggcaacatacct | 6FAM- tgtctgcggcgtttt -MGB |
| HSV1/HSV2 | F-cgcatcaagaccacctcctc R-gtcagctcgtgrttctg | HSV1-Cy5-tggcaacgcggcccaac-iowaBK |
|  |  | HSV2-6FAM-cggcgatgcgccccag-iowaBK |
| VZV | F-tcactaccagtcatttctatccatctg R-gaaaacccaaaccgttctcgag | HEX-tgtctttcacggaggcaaacacgt-iowaBK |
| EBV | F-ctgggcaaggagctgtttg R-ggccgcttgtaaaattgca | 6FAM-ctcggctgtggagcaggctt-iowaBK |
| HHV6 | F-gaagcagcaatcgcaacaca R-acaacatgtaactcggtgtacggt | Cy5-aacccgtgcgccgctccc-iowaBK |
| HHV7 | F-cggaagtcactggagtaatgacaa R-ccaatccttccgaaaccgat | HEX-ctcgcagattgcttgttggccatg-iowaBK |
| HHV8 | F-cctgtcctctggtccccat R-atcgttgcctatttctttttgcc | HEX-ccggcgtcagacattctcacaacc-iowaBK |
| CMV | F-tcgcgcccgaagagg R-cggccggattgtggatt | Cy5-caccgacgaggattccgacaacg-iowaBK |
| Parvovirus B19 | F-gggtttcaagcacaagYagtaaaaga R-cggYaaacttccttgaaaatg | 6FAM-cagctgcccctgtgg-MGB |
| BKV | F-ggaaagtctttagggtcttctaccttt R-gatgaagatttattytgccatgarg | 6FAM-atcactggcaaacat-MGB |
| JCV | F-ggaaagtctttagggtcttctaccttt R-gaagacctgttttgccatgaaga | 6FAM-atcactggcaaacat-MGB |
| GAPDH | F-tgtgctcccactcctgatttc R-cctagtcccagggctttgatt | 6FAM-aaaagagctaggaaggacaggcaacttggc-iowaBlack |

| RNA virus | Primer sequence | Probe sequence |
| --- | --- | --- |
| HIV-1 | F1- ggacatcaagcagcYatgcaaatg F2- ggacaccaRgcagctatgcaaatg R1- tgctatRtcacttccccttggttctct R2- tgctatatcacttcccctaggttccct R3-tgctatatcactccccctaggttctct | 6FAM-acHatcaatgaggaagctgcagaa-MGB |
| HIV-2 | F-gcaggtagagcctgggtgttc R-cttgcttctaaYtggcagctttatt | 6FAM-tgggcagaYggctccacgc-BHQ |
| HTLV-1 | F-ggccacctgtccagagca R-actgtagagctgagccgataacg | 6FAM-Mtcacctgggaccccatcgatgga-BHQ |
| HTLV-2 | F-ggccacctgtccagagca R-attggagaggagagctgacaacg | 6FAM-Mtcacctgggaccccatcgatgga-BHQ |
| HCV | F-gtctagccatggcgttagta R-ctcgcaagcaccctatcaggcagt | 6FAM-ctgcggaaccggtgagtacac-BHQ |
| WNV | F-cagtcacaagtgaatgctta R-ctggcatgctgatcttg | 6FAM-cacctcttgcgaaggacctc-BHQ |
