# Supplementary material for: High-sensitivity virus and mycoplasma screening test reveals high prevalence of parvovirus B19 infection in human synovial tissues and bone marrow
Source: Stem Cell Res Ther. 2018 Mar 27;9:80. doi: 10.1186/s13287-018-0811-7 (PMC5870688; doi:10.1186/s13287-018-0811-7)
Supplement: Supplementary file 3 — Table S2. Primer and probe sequence of nested PCR analysis and sequencing for parvovirus B19 virus genome. (DOCX 14 kb) [file 13287_2018_811_MOESM3_ESM.docx]

| Supplementary table 2. Primer and Probe Sequence of nested PCR analysis and sequencing for Parvovirus B19 virus genome | | | |
| --- | --- | --- | --- |
| Target: NS1-VP1u region (1167-nt) | | | |
|  | name | sequence (5’-3’) | Final conf. (μM) |
| 1st PCR | parvoB19-1F | ggcatggttaaYtggaataa | 0.2 |
|  | parvoB19gt-1R2-1 | aaagtggccccctcactccacat | 0.1 |
|  | parvoB19gt-1R2-2 | aatgtagccccttcattccacat | 0.1 |
| 2nd PCR | parvoB19secondF | ctgggatgaaggYattattaagtc | 0.2 |
|  | parvoB19gt-1R1 | gcagaRttaactgaagtcatgct | 0.2 |
| Sequence primer | parvoB19secondF | ctgggatgaaggYattattaagtc | 0.16 |
|  | parvoB19-2F2 | gtaagatgYagccctgacatg | 0.16 |
|  | parvoB19-3F2 | gtgaactgttagtWggggttga | 0.16 |
|  | parvoB19-4F2-1 | tctctgtttgacttagttgctcg | 0.16 |
