# Supplementary material for: High-sensitivity virus and mycoplasma screening test reveals high prevalence of parvovirus B19 infection in human synovial tissues and bone marrow
Source: Stem Cell Res Ther. 2018 Mar 27;9:80. doi: 10.1186/s13287-018-0811-7 (PMC5870688; doi:10.1186/s13287-018-0811-7)
Supplement: Supplementary file 4 — Table S3. Primer and probe sequence of nested PCR analysis and sequencing for HSV-1, CMV, and parvovirus B19 mRNAs. (DOCX 14 kb) [file 13287_2018_811_MOESM4_ESM.docx]

| Supplementary table 3. Primer and Probe Sequence of nested PCR analysis and sequencing for HSV-1, CMV, Parvovirus B19 mRNAs | | |
| --- | --- | --- |
| Virus mRNA | Primer sequence | Probe sequence |
| HSV-1 ICP4 immediate early | F-cgtggggtccggatacg | 6FAM-agtacgacgacgcagccgac-BHQ |
|  | R-acgacgatgacggggac |  |
| HSV-1 gricoprotein B | F-cgcatcaagaccacctcctc | 6FAM-tggcaacgcggcccaac-BHQ |
|  | R-gtcagctcgtgRttctg |  |
| CMV immideate ealy | F-ccaaggtgccacggcc | 6FAM-tgaccaaggccacgacgttcctg-BHQ |
|  | R-tgttaacctccttcctcaacatagtct |  |
| CMV UL89 Late gene | F-cagcagcaaRtggaagttttgt | 6FAM-ctcggatgctgttggtgttgta-BHQ |
|  | R-ggcgctttttgccagttg |  |
| Parvovirus B19 | F-gggctgctttttcctggact | 6FAM-ccccgcgctctagtacgcccat-BHQ |
|  | R-gggcttccgacaaatgattc |  |
