# Supplementary material for: High-sensitivity virus and mycoplasma screening test reveals high prevalence of parvovirus B19 infection in human synovial tissues and bone marrow
Source: Stem Cell Res Ther. 2018 Mar 27;9:80. doi: 10.1186/s13287-018-0811-7 (PMC5870688; doi:10.1186/s13287-018-0811-7)
Supplement: Supplementary file 5 — Figure S2. Primer and probe design for quantitative PCR analysis for virus multi-spliced mRNA. (A) Parvovirus B19. (B) CMV. (C) HSV-1. (PPTX 135 kb) [file 13287_2018_811_MOESM5_ESM.pptx]

## Slide 1
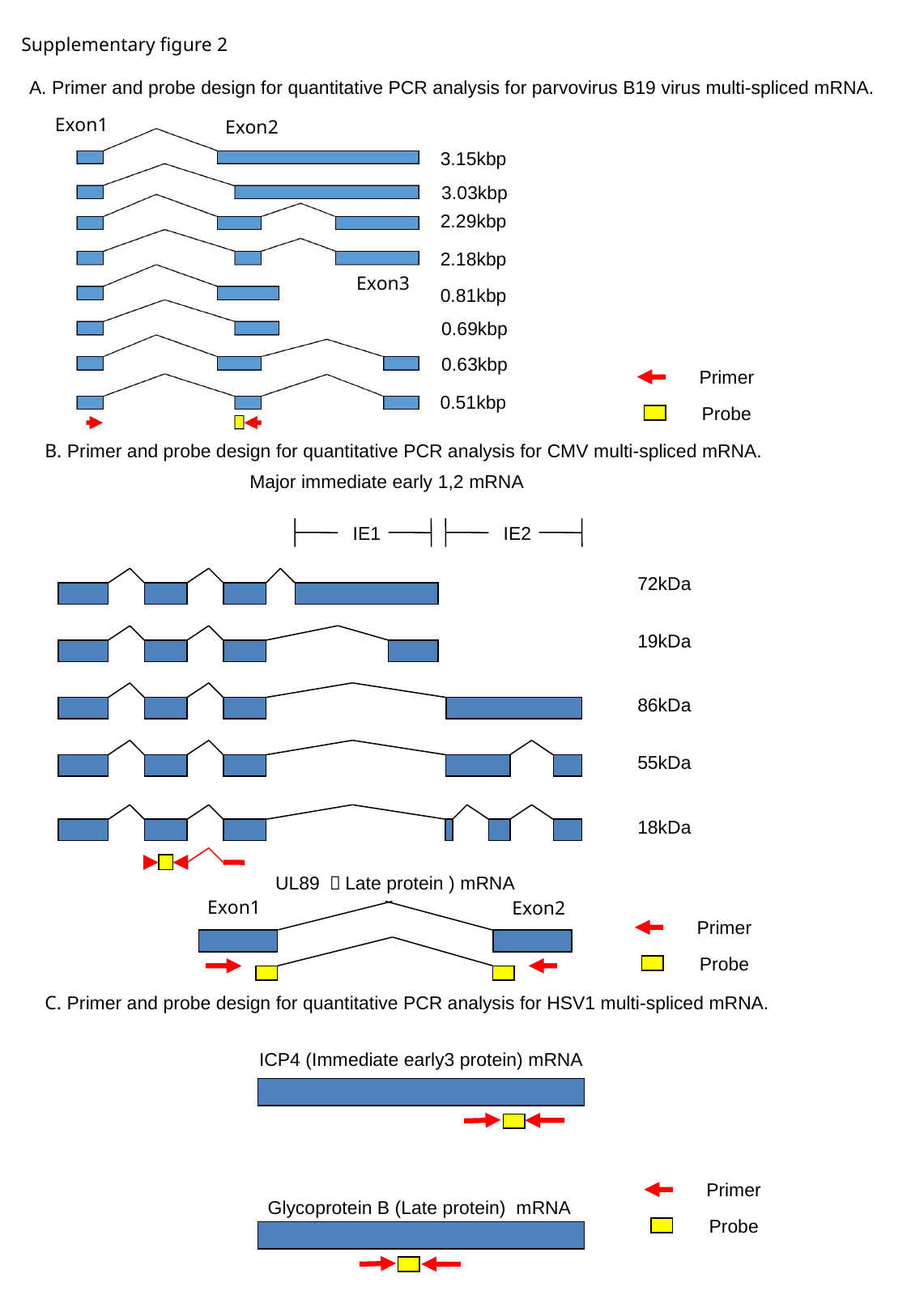

Supplementary figure 2
A. Primer and probe design for quantitative PCR analysis for parvovirus B19 virus multi-spliced mRNA.
Exon1
Exon2
3.15kbp
3.03kbp
2.29kbp
2.18kbp
0.81kbp
0.69kbp
0.63kbp
0.51kbp
Exon3
Primer
Probe
B. Primer and probe design for quantitative PCR analysis for CMV multi-spliced mRNA.
Major immediate early 1,2 mRNA
IE1
IE2
72kDa
19kDa
86kDa
55kDa
18kDa
ex1
ex2
UL89 （Late protein ) mRNA
Exon1
Exon2
Primer
Probe
C. Primer and probe design for quantitative PCR analysis for HSV1 multi-spliced mRNA.
ICP4 (Immediate early3 protein) mRNA
Primer
Glycoprotein B (Late protein) mRNA
Probe
